# Genome-Wide Interaction Study of Late-Onset Asthma With Seven Environmental Factors Using a Structured Linear Mixed Model in Europeans

**DOI:** 10.3389/fgene.2022.765502

**Published:** 2022-03-30

**Authors:** Eun Ju Baek, Hae Un Jung, Tae-Woong Ha, Dong Jun Kim, Ji Eun Lim, Han Kyul Kim, Ji-One Kang, Bermseok Oh

**Affiliations:** ^1^ Department of Biomedical Science, Graduate School, Kyung Hee University, Seoul, Korea; ^2^ Department of Biochemistry and Molecular Biology, School of Medicine, Kyung Hee University, Seoul, Korea

**Keywords:** asthma, late-onset asthma, genome-wide interaction study, structured linear mixed model, environmental factor

## Abstract

Asthma is among the most common chronic diseases worldwide, creating a substantial healthcare burden. In late-onset asthma, there are wide global differences in asthma prevalence and low genetic heritability. It has been suggested as evidence for genetic susceptibility to asthma triggered by exposure to multiple environmental factors. Very few genome-wide interaction studies have identified gene-environment (G×E) interaction loci for asthma in adults. We evaluated genetic loci for late-onset asthma showing G×E interactions with multiple environmental factors, including alcohol intake, body mass index, insomnia, physical activity, mental status, sedentary behavior, and socioeconomic status. In gene-by-single environment interactions, we found no genome-wide significant single-nucleotide polymorphisms. However, in the gene-by-multi-environment interaction study, we identified three novel and genome-wide significant single-nucleotide polymorphisms: rs117996675, rs345749, and rs17704680. Bayes factor analysis suggested that for rs117996675 and rs17704680, body mass index is the most relevant environmental factor; for rs345749, insomnia and alcohol intake frequency are the most relevant factors in the G×E interactions of late-onset asthma. Functional annotations implicate the role of these three novel loci in regulating the immune system. In addition, the annotation for rs117996675 supports the body mass index as the most relevant environmental factor, as evidenced by the Bayes factor value. Our findings help to understand the role of the immune system in asthma and the role of environmental factors in late-onset asthma through G×E interactions. Ultimately, the enhanced understanding of asthma would contribute to better precision treatment depending on personal genetic and environmental information.

## Introduction

Asthma is one of the most common chronic diseases worldwide (Beasley et al., 2015). The global prevalence of asthma in adults is estimated as 4.3%, with wide variation by as much as 21-fold among countries (Bhandaru et al., 2009; Akinbami et al., 2012). Despite the 42% reduction in asthma-related death rates worldwide between 1990 and 2013, no therapeutic regimen is known, and public health efforts to assess and manage asthma remain limited (Ahn et al., 2015). Therefore, it is critical to better understand risk factors such as genes or the environment that are implicated in asthma.

Asthma is a heterogeneous disease in both children and adults. Dissecting the heterogeneity of asthma could reveal the subtype-specific phenotypes and pathogenesis and lead to the development of new therapeutic strategies. In the classification of asthma based on the age of onset, late-onset asthma refers to onset as early as 12 years of age or as late as over 65 years (Amelink et al., 2013) and differs from early-onset asthma in many characteristics. Late-onset asthma is more heterogeneous and severe, often non-atopic, and frequently associated with a faster decline in lung function, partially because of underdiagnosis compared to early-onset asthma, suggesting more accelerated disease or prolonged asymptomatic periods without treatment (Amelink et al., 2013; Papi et al., 2018; Kaur and Chupp, 2019). Several twin studies suggested a low genetic contribution to late-onset asthma predisposition, with heritability estimates of as much as 57–73% in adults, compared to 82% in children (Thomsen et al., 2010; Ullemar et al., 2016). Genetic heritability explained by common variants is also lower for late-onset asthma (onset at ages between 20 and 60 years; *h* ^2^_g_ = 10.6%) than for early-onset asthma (onset at ages between 0 and 19 years; *h* ^2^_g_ = 25.6%), and the genetic correlation (*r*_g_) between late-onset and early-onset asthma is 0.67 (Ferreira et al., 2019). The lower heritability estimated by variants (*h* ^2^_g_) than that by twin studies indicates that hypothesis-free approaches, such as genome-wide association studies of previously unknown genetic loci associated with asthma, should be used.

Studies of the association between the migration status and asthma prevalence provide insight into the importance of environmental factors in the development of asthma (Cabieses et al., 2014). The prevalence of asthma is lower in immigrants from countries with low prevalence than in natives of the host country with a high prevalence, although these values reach similar proportions with an increasing time of residence (Cabieses et al., 2014). Early-onset asthma is likely attributable to atopy and potentially genetic factors, whereas late-onset asthma appears to be related to environmental risk factors, including physical inactivity, alcohol consumption, sleep disorder, sedentary behavior, mental health, and socioeconomic status (Vally et al., 2000; Hedlund et al., 2006; Eijkemans et al., 2012; Meltzer et al., 2014; Tan et al., 2015; Vancampfort et al., 2017; Kavanagh et al., 2018; Najjab et al., 2020). In late-onset asthma, global differences in asthma prevalence may be attributed to interactions of an individuals’ genetic susceptibility with multiple environmental exposures (von Mutius, 2009). Most G×E interaction studies of asthma have applied a candidate gene approach, preventing identification of unknown G×E interactions (Morales and Duffy, 2019). G×E interactions for late-onset asthma have been studied to determine whether previously known loci interact with smoking, NO_2_, gas cooking, or endotoxin exposures (Blomstrom-Lundqvist et al., 2003; Cesarovic et al., 2011; Amaral et al., 2014; Miyake et al., 2014; Bowatte et al., 2017).

Very few genome-wide interaction studies have focused on G×E interaction loci for asthma susceptibility in adults (Turner, 2017; Hernandez-Pacheco et al., 2019; Morales and Duffy, 2019). A genome-wide interaction analysis of active tobacco smoking in 4,057 patients with adulthood-onset asthma of European ancestry was performed using data from the GABRIEL consortium (Demenais et al., 2018). Intergenic single-nucleotide polymorphisms (SNPs) (rs9969775 in *MPDZ-NFIB*, odds ratio, OR = 0.50, *p* = 7.63 × 10^–5^; rs5011804 in *KRAS-IFLTD1*, OR = 1.50, *p* = 1.21 × 10^–4^) were suggested to interact with active tobacco smoking for late-onset asthma with potential regulatory functions linked to gene expression regulation in the lung tissue (Demenais et al., 2018). Another study examined the interaction of genetic variants with age on the response to inhaled corticosteroids (measured by the occurrence of exacerbations) in 1,321 adult and child patients who had asthma and were of European ancestry (Dahlin et al., 2020). Two SNPs (rs34631960 in *THSD4*, OR = 2.3, *p* = 3.64 × 10^–8^; rs2328386 in *HIVEP2*, OR = 0.5, *p* = 4.98 × 10^–8^) were identified as significant pharmacogenomics loci on asthma by joint analysis of genome-wide interaction results from discovery and replication populations (Dahlin et al., 2020).

This study was conducted to identify loci of late-onset asthma susceptibility showing G×E interactions with multiple environments. We conducted gene-by-single environment (G×singleE) interaction analyses using a fixed effect logistic regression model, PLINK (Purcell et al., 2007) and gene-by-multi-environment (G×multiE) interaction analysis using a structured linear mixed model, StructLMM (Moore et al., 2019). Of the various environmental factors associated with asthma, seven factors were selected for G×E interaction analyses based on relatedness, including alcohol intake frequency, body mass index (BMI), insomnia, metabolic equivalent of task (MET) score, neuroticism score, time spent watching TV, and the Townsend deprivation index (TDI) (Vally et al., 2000; Hedlund et al., 2006; Eijkemans et al., 2012; Meltzer et al., 2014; Vancampfort et al., 2017; Najjab et al., 2020).

## Materials and Methods

### Study Population and Design

The United Kingdom Biobank (UKB) resource was used for the discovery set of G×singleE and G×multiE interaction analyses, and for one replication set of G×multiE interaction analysis. The UKB is a population-based cohort that recruited over 487,409 individuals aged 40–69 years in the United Kingdom during 2006–2010 (Collins, 2012). The initial assessment visit was finished during 2006–2010 (baseline instance 0) and the repeat assessment (or supplemental assessment) visits were followed by the periods 2012–2013 (instance 1), 2014–2018 (instance 2), and 2019+ (instance 3) (Bycroft et al., 2018) The largest subset of participants attended the initial assessment (instance 0) and the other subset completed repeat assessments (instance 1–3) following the initial assessment, and the another subset attended only the supplemental assessments (instance 1–3) without the initial assessment visit. For quality control of the samples, we used the following filter parameters of the Neale lab (https://github.com/Nealelab/UK_Biobank_GWAS): principal component (PC) analysis calculation filter for selecting unrelated samples; sex chromosome filter for removing aneuploidy; filtering of PCs for European sample selection for determining British ancestry; and filters for selecting self-reported ‘white British,’ “Irish,” and “White”. The total unrelated European participants amounted to 356,536.

All participants provided signed consent to participate in the UKB (Biobank, 2007). The UKB has been granted ethical approval to collect participant data by the North West Multicentre Research Ethics Committee, which covers the United Kingdom; National Information Governance Board for Health and Social Care, which covers England and Wales; and Community Health Index Advisory Group, which covers Scotland. The UKB possesses a generic Research Tissue Bank approval granted by the National Research Ethics Service (http://www.hra.nhs.uk/), which allows applicants conduct research on UKB data without obtaining separate ethical approvals. Access to UKB data was granted under application no. 56987: “Classification of asthma patients and identification of group-specific genetic variants”.

**FIGURE 1:**
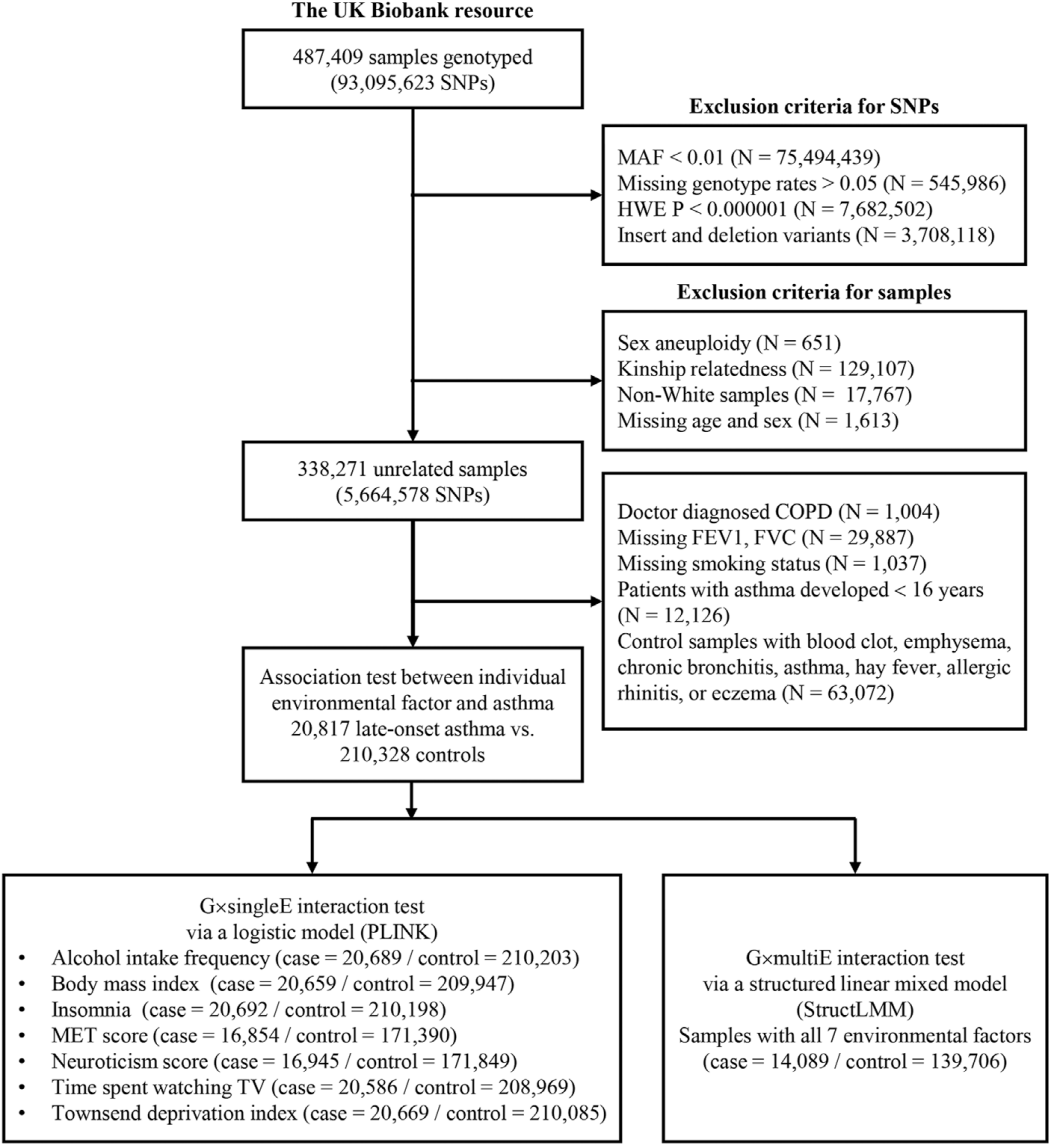
Study design for G×E interaction analysis in the UKB. This diagram includes exclusion criteria for quality control of SNPs and samples. The lower box in the left indicates sample numbers for G×singleE interactions with individual environmental factor on late-onset asthma, whereas the lower box in the right indicates those for G×multiE interactions with seven factors.

For the discovery analysis, we used unrelated Europeans 338,271 with the health information that were collected during the initial assessment period (2006–2010; instance 0). Cases with late-onset asthma (*N* = 20,817) were determined as those that had been diagnosed with asthma by a doctor and had checked for age of onset (≥16 years). Participants who had been diagnosed with chronic obstructive pulmonary disease (COPD) were excluded from the study. Controls (*N* = 210,328) had not been diagnosed with asthma, rhinitis, eczema, hay fever, allergy, emphysema/chronic bronchitis, and COPD (Figure 1). To determine the difference between the case and control groups, those with missing values for forced expiratory volume in 1-s (FEV1) and forced vital capacity were excluded. Participants with missing values for smoking status as covariance were also excluded.

### Environmental Factors

Environmental factors were collected via self-reported touchscreen questionnaires or physical measurements taken manually or index-based on the preceding national census output areas in the UKB. Of the various environmental factors, seven factors were included for G×E interaction analyses based on their relatedness with asthma, including alcohol intake frequency, BMI, insomnia, MET score, neuroticism score, time spent watching TV, and TDI (Vally et al., 2000; Hedlund et al., 2006; Eijkemans et al., 2012; Meltzer et al., 2014; Vancampfort et al., 2017; Najjab et al., 2020).

To confirm the relatedness between environmental factors and asthma, we used a logistic regression model. “Prefer not to answer” and “I don’t know” were set to “missing” in our analysis. Alcohol intake frequency was encoded as: 1 = “daily or almost daily”, 2 = “three or four times a week”, 3 = “once or twice a week”, 4 = “one to three times a month”, 5 = “special occasions only”, or 6 = “never”. BMI was calculated in units of kg/m^2^ from the height and weight measured during the initial assessment center visit (Rask-Andersen et al., 2017). Sleeplessness or insomnia complaints were encoded as: 1 = “never/rarely”, 2 = “sometimes”, or 3 = “usually”. The MET value, a measure of physical activity, was derived from the work undertaken during the International Physical Activity Questionnaire which covers the frequency, intensity, and duration of vigorous, moderate, and walking activities (Craig et al., 2003). Time spent in vigorous, moderate, and walking activities was weighted by the energy expended for these categories of physical activities, which are referred to as “total physical activity”. The rules for data processing published by the IPAQ were followed (Committee, 2005). The neuroticism score, a measure of the mental status, reported in UKB was based on 12 neurotic domains from Eysenck Personality questionnaire and was externally derived by Smith et al. (2013) (Smith et al., 2013). Time spent watching TV, a measure of sedentary behavior, represented hours per day; less than 0.5 h was recorded as 0.5. TDI is a composite score for socioeconomic status that is generated for each national census output area and incorporates area inhabitants’ unemployment rates, car- and house-ownership, and the number of people in a household. A higher TDI corresponds to a larger degree of social deprivation. For analysis, individual environmental factors were transformed to a normal distribution using a Gaussian function in StructLMM. The distributions of raw (pre-gaussianized) phenotypes and processed (post-gaussianized) phenotypes are shown in Supplementary Figure S1.

### Genotype Data

At baseline, imputation data of 93, 095, 623 SNPs were available for 487,409 participants using the UKB Axiom Array and the United Kingdom BiLEVE Axiom Array from Affymetrix (Santa Clara, CA, United States ) (Sudlow et al., 2015). Genotyping imputation was performed using the United Kingdom10K Project and 1,000 Genome Project Phase 3 reference panels (Consortium et al., 2015; Genomes Project et al., 2015). Quality control was performed based on the following exclusion criteria using PLINK v.1.90: SNPs with missing genotype call rates >0.05, minor allele frequency < 0.01, and *P* for Hardy-Weinberg equilibrium test <1.00 × 10^–6^). A total of 5,664,578 SNPs was retained for further analysis.

### Statistical Analysis

We performed association analysis, correlogram, Manhattan, bar, and violin plottings, one-way analysis of variance, and correlation analysis (Spearman’s rank correlation) using the R stats package (version 4.0.2; www.r-project.org). To draw the Manhattan plots, the “qqman” package was used; “corrplot” and “ggplot2” were used to prepare correlogram and violin plots, respectively. Analysis of variance and association analysis were performed using the R stats package.

We used PLINK v.1.90 (Purcell et al., 2007) for SNP quality control. The effects of G×singleE interactions on late-onset asthma for seven individual environmental factors were also analyzed using PLINK v.1.90, adjusted for age, sex, genotyping array, smoking status, and PC1-10. For G×multiE interactions and to calculate the Bayes factor (BF) for individual environmental factors, a structured linear mixed model, StructLMM was performed using the python 3 language (Moore et al., 2019). The classical logistic regression model considers the effect of an environmental factor on genetic interaction as fixed effect while the StructLMM calculates the effect of multiple environments through their combinatory action on genetic interaction as random effect. StructLMM has several strengths: the robustness of power and simultaneous incorporation of multiple environmental factors. Moreover, the BF in StructLMM can be used to assess which environmental factor contributes to its genetic interaction. The BF in StructLMM is a statistical method that compares two models, one with environmental factors and the other without environmental factors, to assess which model is better and to quantify its power.

Of SNPs that were identified in the discovery analysis, independent SNPs were selected through linkage disequilibrium clumping (*p* < 5.00 × 10^–8^ for genome-wide significant regions; *p* < 1.00 × 10^–6^ for genome-wide suggestive regions; *r* ^2^ > 0.01, 1-Mbp boundary distance; PLINK v.1.90).

### Replication

We used two different cohorts for replication of the G×multiE test with three novel SNPs; the first cohort is the European samples of UKB that were not used in the discovery analysis and the second one is the Korean samples from the Health Examinees (HEXA) population-based cohort obtained from the Korean Genome and Epidemiology Study (KoGES)) (Kim et al., 2017).

For the first set of replication in the Europeans of UKB that were unused in the discovery analysis, we excluded 164,969 used participants from the unrelated samples 356,536 with European-British background. Among the remaining samples (*N* = 191,567), cases with late-onset asthma (*N* = 513) were determined as those that had been diagnosed with asthma by a doctor and had checked for age of onset (≥16 years). Participants who had been diagnosed with chronic obstructive pulmonary disease (COPD) were excluded from the study. Controls (*N* = 104,188) had not been diagnosed with asthma, rhinitis, eczema, hay fever, allergy, emphysema/chronic bronchitis, and COPD (Supplementary Figure S2).

In addition, for the replication test, we excluded environmental factors that showed low BF (< 1) at the discovery test for three novel SNPs to spare samples per SNP. Among seven, the following environmental factors for each SNP were included in the replication as follows: for rs117996675, four factors (alcohol intake frequency, BMI, MET score, and time spent watching TV); for rs345749, five factors (alcohol intake frequency, BMI, insomnia, neuroticism, and time spent watching TV); and for rs17704680, four factors (alcohol intake frequency, BMI, TDI, and time spent watching TV). Following exclusion of samples with missing information of environmental factors, the final sample size for rs117996675 was 57,921 (asthma case: control = 379: 57,542); for rs345749 was 57,817 (asthma case: control = 395: 57,422); and for rs17704680 was 101,791 (asthma case: control = 507 : 101,284).

For the second set of replication, we used the Health Examinees (HEXA) population-based cohort obtained from the Korean Genome and Epidemiology Study (KoGES) (Kim et al., 2017). The KoGES HEXA study included participants aged ≥40 years who visited the institutions. Data were collected from 39 sites from 2004 to 2013, and follow-up data were obtained from 2012 to 2016. This study was conducted with bioresources from the National Biobank of Korea and the Center for disease Control and Prevention, Republic of Korea (KBN-2020–017). Among the 58,700 participants, we excluded missing data on the history of asthma, onset age of asthma, COPD, chronic bronchitis, and allergic disease. For the control group, we excluded patients with a history of asthma, onset age of asthma, COPD, chronic bronchitis, and allergic diseases. We excluded patients with COPD and patients who developed asthma before 16 years of age from the case group. For consistency with the UKB samples, we also excluded samples with <40 or >69 years of age when assessed from KoGES HEXA participants and samples with missing information for height, weight, total alcohol intake frequency, psychological well-being index (PWI) short form, and total household income. Finally, 603 patients with late-onset asthma and 36,587 controls were included in the analysis. We used 4 environmental factors: total alcohol intake frequency (preprocessed from soju, beer, makgeolli, jeongjong, wine, liquor, fruit wine, and other alcoholic beverages), BMI, PWI short form, and total household income (Goldberg and Hillier, 1979; Choi et al., 2019; Kim et al., 2019). A detailed schematic illustration of the participant selection process and the characteristics of the selected participants are summarized in Supplementary Figure S3.

### Functional Annotation Tools

We used several approaches to evaluate the functional relevance of the identified SNPs. Haploreg (v.4.1) was used to search for the effect of the identified SNPs on the transcription factor-binding site motif and to perform enhancer enrichment analysis (Ward and Kellis, 2012). Combined Annotation-Dependent Depletion (CADD) was used to evaluate the deleteriousness of SNPs (Rentzsch et al., 2019). The GTEx (v8) database was used to evaluate the association of genetic variation with the expression of genes (Consortium, 2020). PhenoScanner (v2) was further used to search for an association of genetic variants with a broad range of phenotypes (Kamat et al., 2019). Data available from the United States National Institutes of Health Roadmap Epigenomics Program were used to study epigenetic marks such as DNA methylation, histone modifications, and chromatin accessibility and ultimately to understand the role of genetic variation in modulating transcriptional enhancers and promoters (Roadmap Epigenomics et al., 2015).

## Results

### Associations Between Late-Onset Asthma and Environmental Factors

The basic characteristics of UKB participants in this study are described for G×singleE interaction (*N* = 231,145) and for G×multiE interaction analyses (*N* = 153,795), respectively in Table 1 and Supplementary Table S1, (Figure 1). Cases with late-onset asthma had been doctor-diagnosed at ≥16 years old. Female frequency and smoking status differed significantly between asthma cases and controls, and FEV1 and the FEV1 per forced vital capacity ratio were significantly higher in late-onset asthma cases than in controls (Table 1).

**TABLE 1:** Characteristics of late-onset asthma cases and controls for StructLMM analysis from the UKB.

		Asthma case (*N* = 14,089)	Control (*N* = 139,706)
Age (years) *		55.80 ± 8.08	56.68 ± 7.94
Sex (%)^†^	Female	8,597 (61.02%)	70,112 (50.19%)
	Male	5,492 (38.98%)	69,594 (49.81%)
Onset age (years)		39.8 ± 12.84	-
Smoking status (%)^†^	Current smoker	1,083 (7.31%)	13,909 (9.56%)
	Previous smoker	5,336 (36.03%)	49,281 (35.27%)
	Never smoker	7,670 (51.79%)	76,516 (54.77%)
FEV1% predicted *		83.73 ± 17.54	90.42 ± 16.38
FEV1/FVC ratio *		0.74 ± 0.08	0.76 ± 0.07
Alcohol intake frequency (%)^†^	Daily or almost daily	1,048 (7.08%)	8,119 (5.81%)
	Three or four times a week	1,618 (10.93%)	12,886 (3.22%)
	Once or twice a week	1,634 (11.03%)	14,740 (10.55%)
	One to three times a month	3,514 (23.73%)	37,428 (26.79%)
	Special occasions only	3,320 (22.42%)	35,044 (25.08%)
	Never	2,955 (19.95%)	31,489 (22.54%)
BMI (kg/m^2^) *		28.05 ± 5.16	27.18 ± 4.50
Insomnia (%)^†^	Never/rarely	3,110 (22.07%)	66,135 (47.34%)
	Sometimes	6,517 (46.26%)	36,041 (25.80%)
	Usually	4,462 (31.67%)	37,530 (26.86%)
MET score (min/week) *		2,600.27 ± 2,670.44	2,743.63 ± 2,752.71
Neuroticism score *		4.48 ± 3.32	3.88 ± 3.19
Time spent watching TV (hours/day) *		2.77 ± 1.67	2.71 ± 1.57
TDI *		-1.49 ± 2.97	-1.73 ± 2.83

Data, mean ± standard deviation (SD) or n (%), unless otherwise stated; FEV1, forced expiratory volume in 1s; FVC, forced vital capacity.

Student’s t-test is used to compare mean differences of quantitative variables between cases and controls; * denotes a significant difference in mean between cases and controls.

Chi-squared test is used to check for imbalances of categorical variables between cases and controls. † denotes a significant imbalance between cases and controls.

BMI, body mass index; MET, metabolic equivalent of task; TDI, townsend deprivation index.

Of the numerous factors associated with asthma, seven factors were selected for G×E interaction analyses based on relatedness, including alcohol intake frequency, BMI, insomnia, MET score, neuroticism score, time spent watching TV, and TDI (Vally et al., 2000; Hedlund et al., 2006; Eijkemans et al., 2012; Meltzer et al., 2014; Vancampfort et al., 2017; Najjab et al., 2020). We examined the interaction between environmental factors and genetic variants on late-onset asthma. A logistic regression model for association between individual factors and late-onset asthma was used, adjusted for age, sex, and smoking status (Supplementary Table S1). All seven environmental factors were significantly associated with late-onset asthma (*p* < 0.05/7 = 7.14 × 10^–3^) in the following order: BMI (*p* = 1.55 × 10^–124^), neuroticism score (*p* = 8.60 × 10^–62^), insomnia (*p* = 5.30 × 10^–48^), TDI (*p* = 8.79 × 10^–21^), alcohol intake frequency (*p* = 4.74 × 10^–18^), time spent watching TV (*p* = 2.01 × 10^–14^), and MET score (*p* = 3.37 × 10^–5^) (Supplementary Table S2). Categorical variables such as alcohol intake frequency and insomnia showed significant imbalances, whereas quantitative variables including BMI, MET score, neuroticism score, time spent watching TV, and TDI differed significantly between asthma cases and controls (Table 1). Additionally, correlation analyses between pairs of seven environmental factors and late-onset asthma showed that most environmental factors were positively correlated with an individual environmental factor except for the MET score (Supplementary Table S3, Supplementary Figure S3). The MET score was negatively correlated with BMI (*p* = 1.41 × 10^–228^), insomnia (*p* = 8.17 × 10^–32^), neuroticism score (*p* = 1.77 × 10^–67^), and time spent watching TV (*p* = 5.75 × 10^–18^), whereas it was positively correlated with alcohol intake frequency (*p* = 3.31 × 13^–10^) and TDI (*p* = 2.05 × 10^–35^).

### G×singleE Interaction Analysis for Late-Onset Asthma

We performed G×singleE interaction analyses for late-onset asthma using seven environmental factors: alcohol intake frequency, BMI, insomnia, MET score, neuroticism score, time spent watching TV, and TDI in 231,145 individuals of European ancestry. These analyses were conducted using PLINK after adjusting for age, sex, genotyping array, smoking status, and 1–10 PCs. As a result of seven genome-wide G×singleE interaction analyses, 26 SNPs satisfied the genome-wide suggestive level (*p* < 1.00 × 10^–6^), with no SNP satisfying the threshold of genome-wide significance (*p* < 5.00 × 10^–8^/7 = 7.14 × 10^–9^) (Supplementary Table S4). Manhattan plots for each interaction result are depicted in Supplementary Table S4, and the information on the asthma-related genome-wide association study regarding the 26 SNPs is summarized in Supplementary Table S4.

### G×multiE Interaction Analysis for Late-Onset Asthma

**FIGURE 2:**
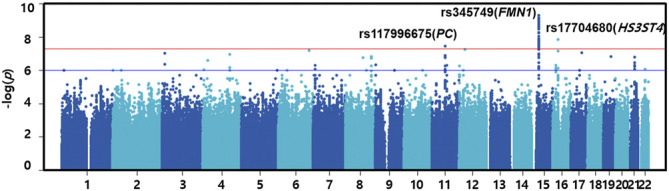
Manhattan plot of G×multiE interaction analysis on late-onset asthma. The red line indicates the threshold of genome-wide significance (*p* = 5.00 × 10^–8^), whereas the blue line indicates a genome-wide suggestive level (*p* = 1.00 × 10^–6^).

**FIGURE 3:**
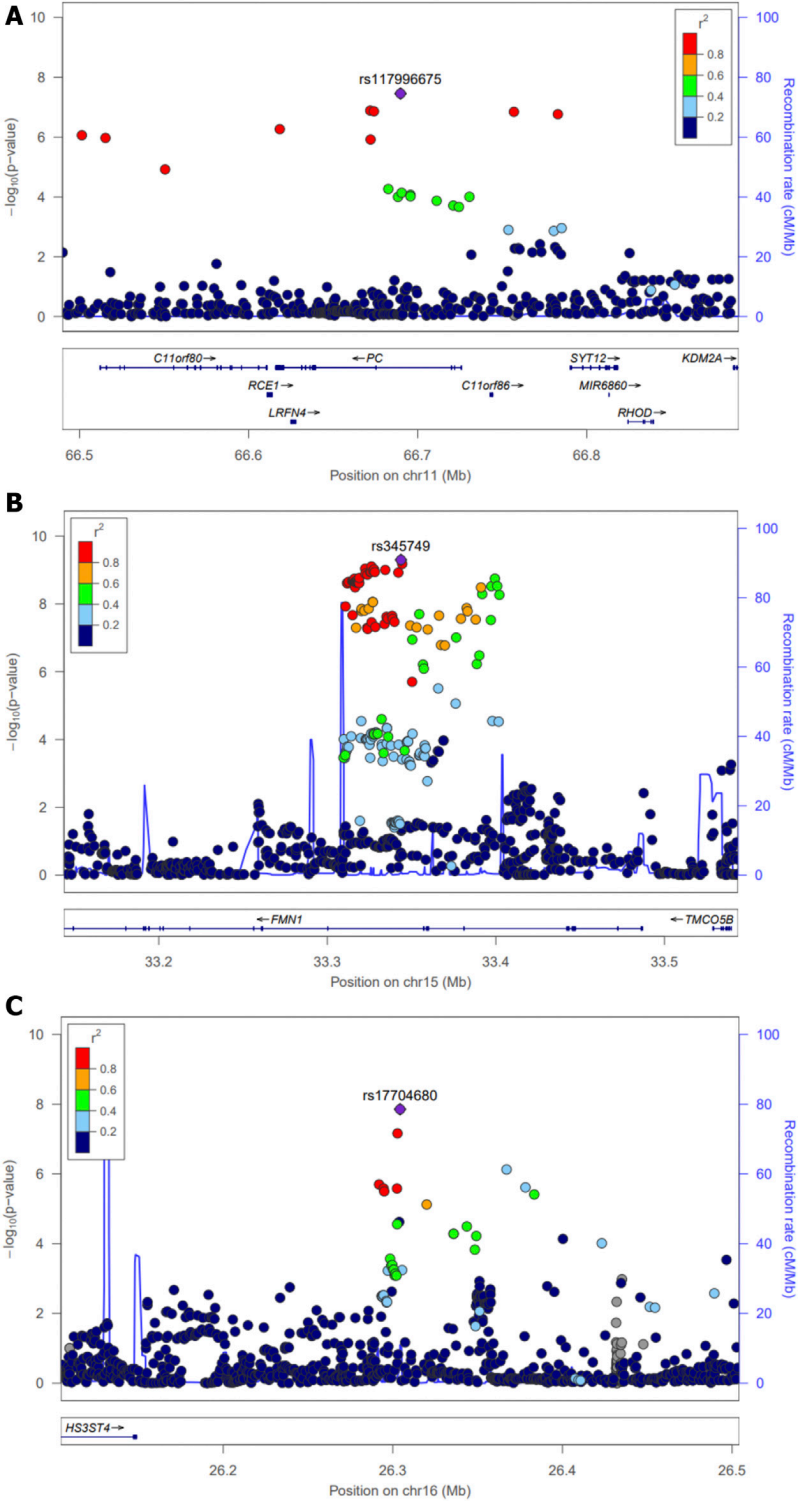
Regional association plots for three novel loci across a 0.5-Mb window. Interaction (*p*-values) of individual SNPs in G×multiE interaction analysis was plotted as −log_10_(*P*) against the chromosomal base pair position (hg19). The *y*-axis on the right shows the recombination rate, estimated from the 1,000 Genomes EUR population. The purple diamonds indicate individual lead SNPs. Regional plots around **(A)** rs117996675 **(B)** rs345749, and **(C)** rs17704680.

**FIGURE 4:**
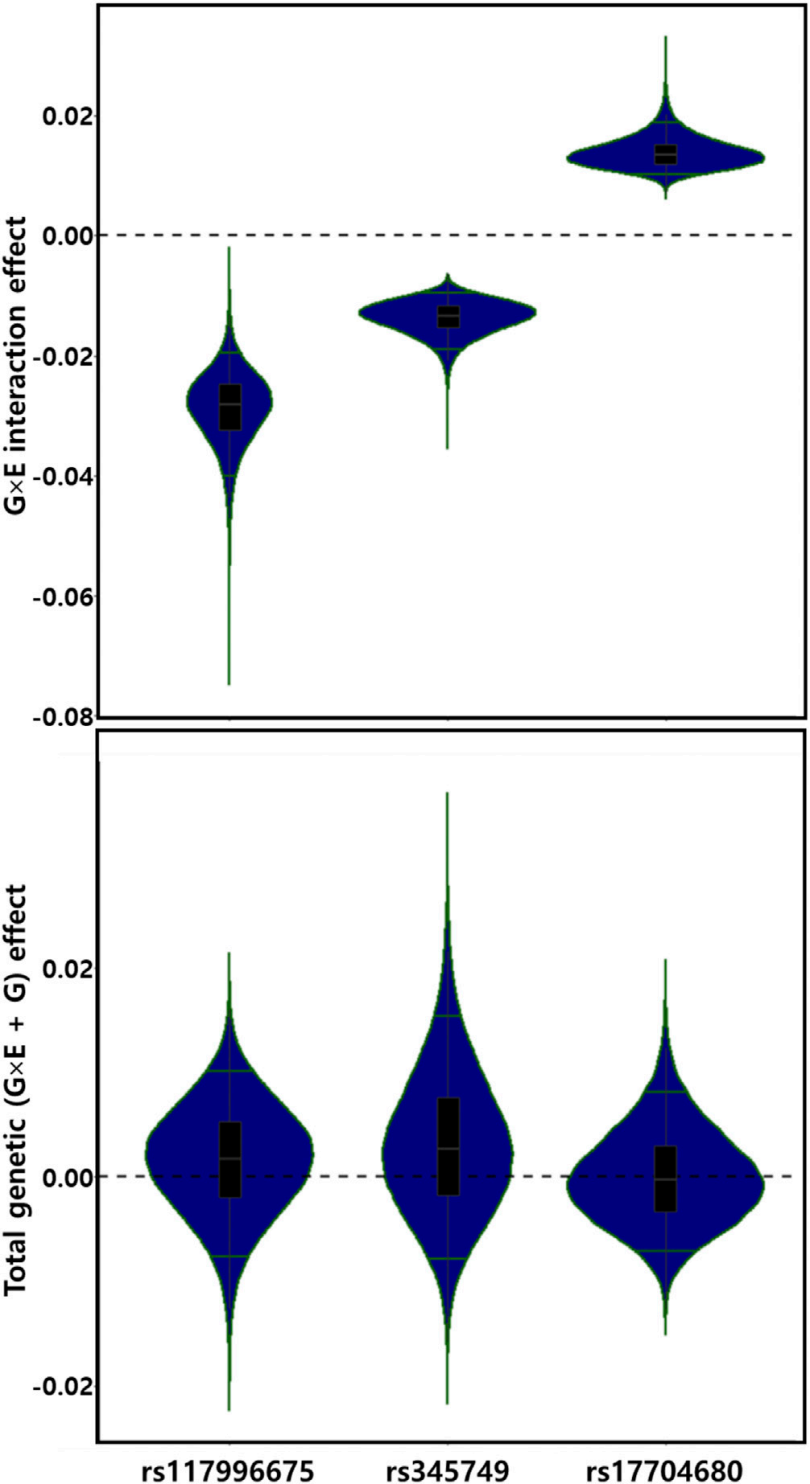
Distributions of G×E interaction and total genetic effects of three genetic alleles. G×E interaction and total genetic (G×E + G) effects are depicted by violin plots. The green line indicates the top and bottom 5% quantiles.

For the discovery set of G×multiE interaction study, we used StructLMM, a structured linear mixed model, for late-onset asthma using seven environmental factors included as multivariate environmental components, adjusted for age, sex, genotyping array, smoking status, and 1–10 PCs, in 153,795 individuals of European ancestry. We identified three independent genome-wide significant SNPs (rs117996675, *p* = 4.25 × 10^–8^; rs345749, *p* = 4.41 × 10^–10^; rs17704680, *p* = 1.26 × 10^–8^) (Table 2) and 17 suggestive SNPs, as shown in the Manhattan plot and regional association plots (Figures 2, 3; Supplementary Table S5). The three genome-wide significant SNPs have not been previously reported as loci associated with asthma or asthma-related traits, such as allergy, eczema, hay fever, and whizzing. We calculated the per-individual effects of the total genetic effect (G + G×E), G×E interaction (G×E), and the main effect (G) of each of the three SNPs, indicating a wider variation of G×E interaction effects than the total genetic effect (Figure 4; Supplementary Table S6) (Purcell et al., 2007; Moore et al., 2019).

**TABLE 2:** G×multiE interaction between SNPs and seven environmental factors on late-onset asthma. We used the italics for human gene names and *P*-values.

SNP	Chromosome	Position^a^	Nearby gene	Minor allele	European MAF (%)	*P*-value^b^
rs117996675	11	66,689,820	*PC*	T	5.219	4.25E-08
rs345749	15	33,343,682	*FMN1*	A	41.44	5.03E-10
rs17704680	16	26,304,247	*HS3ST4*	A	17.54	1.26E-08

a Chromosomal positions are based on the 1,000 Genomes Project’s haplotype phase 1 in NCBI, build 37 (hg19).

b p-values for G×multiE interaction were assessed using a StructLMM, adjusted for age, sex, batch size, smoking status, and PC1-10.

SNP, single nucleotide polymorphism; MAF, minor allele frequency.

### Relevance of Environments in G×multiE Interaction Study for Late-Onset Asthma

To explore which environmental factors are the most relevant to individual G×E interaction signals, we estimated the BF values for each environmental factor using a statistical method that assesses the comparative power of each model comparing between the full model and model without an individual environmental factor (Moore et al., 2019). A BF value less than 1 indicates that the environmental factors do not improve the test power of StructLMM through the G×E interaction.

**FIGURE 5:**
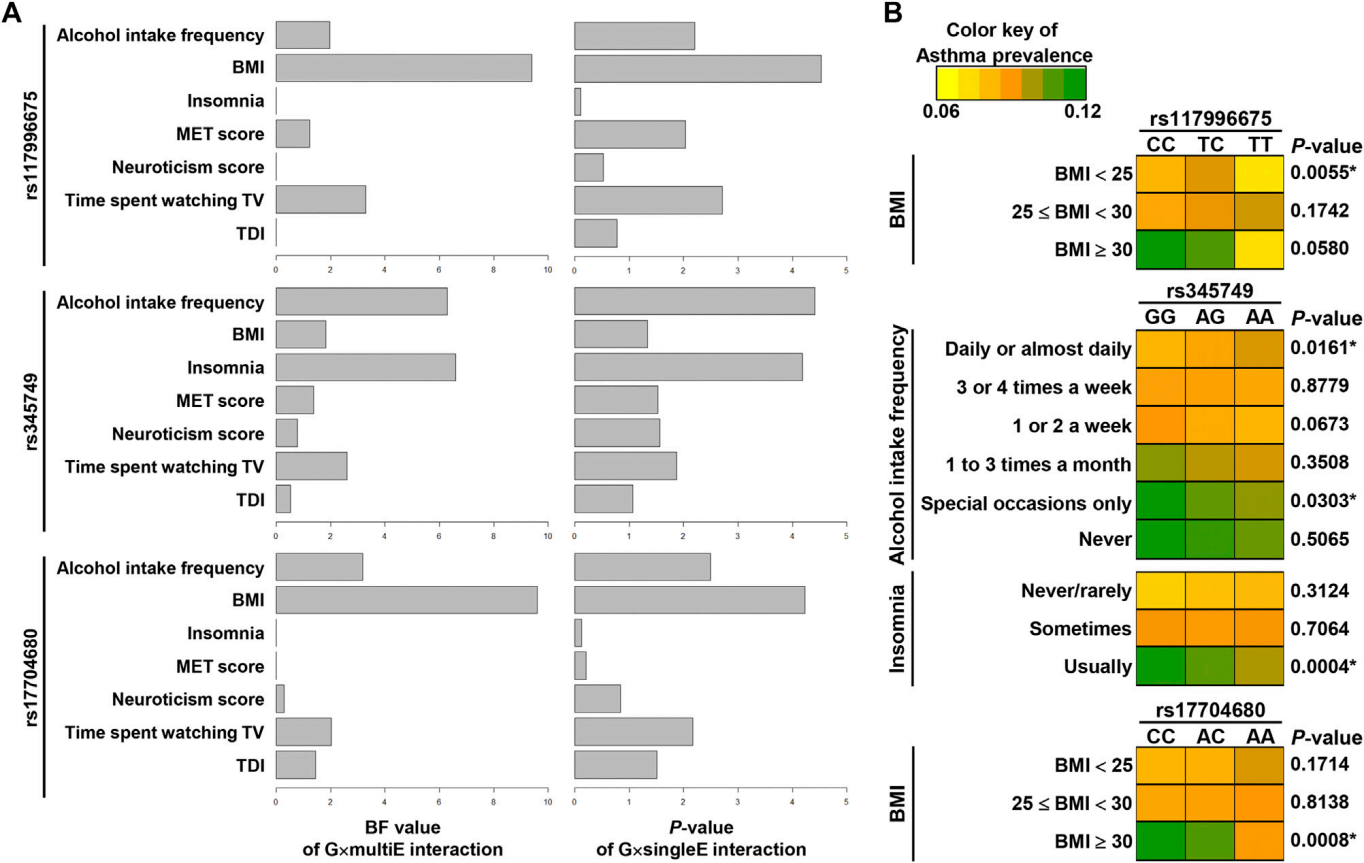
Relevance of environmental factors in G×E interaction effects of three SNPs. **(A)** Graphs in the left show BF values per an environmental factor *via* G×multiE interaction (StructLMM), whereas those in the right show *p*-values [−log_10_(*P*)] by G×singleE interaction analysis for 3SNPs. **(B)** Asthma prevalence was visualized by heat maps, differentiated by alleles of three SNPs. *p*-values are the result of chi-squared test with *p*-values (*) of < 0.05 indicating significance.

The analyses of BF values suggested that for rs117996675 and rs17704680, BMI was the most relevant among the seven environmental factors; for rs345749, insomnia and alcohol intake frequency were the most relevant (Figure 5A; Supplementary Table S7). In addition, the BF values of StructLMM and *p*-values of G×singleE interaction were ranked in the same order or similarly as follows: for rs117996675, BMI > time spent watching TV > alcohol intake frequency > MET score > insomnia, neuroticism score, and TDI; for rs345749, insomnia and alcohol intake frequency > time spent watching TV > BMI, neuroticism score, TDI, and MET score; lastly for rs17704680, BMI > alcohol intake frequency > time spent watching TV > TDI > neuroticism score > MET score, and insomnia (Figure 5A; Supplementary Table S7). The similarity between orders of BF values and *p*-values of G×singleE interaction supports the validity of the interaction power in StructLMM.

To confirm the relevance of environmental factors, we tested whether asthma prevalence differed between alleles of variants within an individual category of the relevant environment. The asthma prevalence differed significantly between alleles of rs117996675 in participants with BMI < 25 and between alleles of rs17704680 in participants with BMI >30, indicating the interacting effects of variants with a specific subset with certain BMI range on asthma (Figure 5B; Supplementary Table S8). For rs345749, its allelic difference in asthma prevalence was significant in participants with usual insomnia, suggesting insomnia as the most relevant environment (Figure 5B; Supplementary Table S8).

### Functional Annotation

Among the three variants, rs117996675 was within the intron of *PC* (11q13.2 of Chr11), rs345749 was within the intron of *FMN1* (15q13.3 of Chr15), and rs17704680 was in an intergenic region near *HS3ST4* (16p12.1 of Chr16) (Supplementary Table S9). Because noncoding variants may influence gene expression, we examined the sentinel SNPs or proxies in high linkage disequilibrium (*r* ^2^ ≥ 0.8) with three loci from publicly available eQTL data sets (GTEx v8) and the pQTL resource (Sun et al., 2018; Consortium, 2020).

Two sentinel SNPs (rs117996675 and rs345749) and their proxies were associated with variable expression of nine genes in one or more tissues (Supplementary Table S9 and Supplementary Table S10). A sentinel SNP (rs117996675 in the 11q13.2) was associated with expression of multiple genes in various tissues including *RIN1*, *BRMS1*, *ACTN3*, CTD-3074O7.5, RP11-867G23.8, and C11orf80, and its proxies (*r* ^2^ ≥ 0.8) were associated with genes such as *B4GAT1* and RP11-867G23.8 in the whole blood, stomach, skeletal muscle, fat, testis, nerve, and pancreas. The other lead SNP, rs345749, was associated with *FMN1* expression in the thyroid, pituitary, and esophagus mucosa, and its proxy (*r* ^2^ ≥ 0.8) was associated with *GOLGA8O* in the skeletal muscle (Supplementary Table S10). The last SNP, rs17704680, and its proxies showed no eQTL signal but had a weak pQTL with thymic stromal lymphopoietin (TSLP) in the blood (Sun et al., 2018).

Because genetic variation may affect the modulation of transcriptional enhancers and promoters, we examined data available from the United States National Institutes of Health Roadmap Epigenomics Program (Arking et al., 2014) (Supplementary Table S9). We focused on the integrative data of 5 asthma-related reference human epigenomes (lung, lung fibroblast, primary T helper cells, and lymphoblastoid cells) that were profiled for histone modification patterns, DNA accessibility, DNA methylation, and RNA expression. The sentinel SNPs, rs117996675 and rs345749, and their proxies (*r* ^2^ ≥ 0.8) were enriched in transcriptional enhancers and promoters with slightly different patterns in 5 epigenomes (Supplementary Table S9), indicating that these variants in noncoding regulatory regions affect gene expression. To assess the deleteriousness of the variants, we obtained the CADD scores (Roadmap Epigenomics et al., 2015; Rentzsch et al., 2019). Notably, 16 SNPs in high linkage disequilibrium (*r* ^2^ ≥ 0.8) with rs345749 exhibited high CADD scores (>5) and 2 SNPs, rs345862 and rs345867, scored 10.06 and 14.64, respectively, suggesting that they are likely pathogenic.

### Replication in European Samples

For the first set of replication in another European samples, we used Europeans of UKB that were not used in the discovery analysis as explained in the Methods. We performed a replication test of 3 novel SNPs (rs117996675, rs345749, and rs17704680) in 104,701 European participants (late-onset asthma case = 513/control = 104,188). Since the sample size was very small, we excluded environmental factors that showed low BF (<1) at the discovery test for each of three novel SNPs (Supplementary Table S7). For rs117996675, four factors (alcohol intake frequency, BMI, MET score, and time spent watching TV) were included in the replication test; for rs345749, included five factors (alcohol intake frequency, BMI, insomnia, neuroticism, and time spent watching TV); and for rs17704680, included four factors (alcohol intake frequency, BMI, TDI, and time spent watching TV) (Supplementary Table S11).

We performed StructLMM using the set of environmental factors for each of three SNPs. Consequently, rs17704680 showed a significant G×multiE interaction signal (*p* = 3.93 × 10^–3^) that passed the threshold of significance (*p* = 1.67 × 10^–2^ = 0.05/3). However, rs117996675 and rs345749 did not showed any significant interaction (*p* = 4.10 × 10^–1^ for rs117996675; *p* = 7.28 × 10^–1^ for rs345749) (Supplementary Table S12). We summarized the results of BF and G×singleE interaction effects of the 3 SNPs in the replication set of UKB (Supplementary Table S13). Among 4 factors, the best BF factor for rs17704680 was attributed to BMI (BF = 3.43), supporting the discovery result using UKB samples with the BF of BMI showing the highest BF value (9.60) (Supplementary Table S13). For rs117996675 and rs345749, BF values were much less than 1for all environmental factors tested.

### Replication in East-Asian Samples

We performed a replication test of 2 SNPs (rs345749 and rs17704680) in 37,460 East-Asian samples (36,857 controls and 603 late-onset asthma cases from the HEXA study) using StructLMM. Female frequency and smoking status differed significantly between cases with late-onset asthma and controls (Supplementary Table S14). For replication in East-Asian individuals, because of the limited availability of the factors surveyed, only four environmental factors were used, including total alcohol intake frequency, BMI, PWI, and income. Total alcohol intake frequency and income differed significantly between asthma cases and controls, whereas BMI and PWI were significantly higher in cases with late-onset asthma than in controls (Supplementary Table S14). The last variant (rs117996675) had a rare allele frequency in East Asians and was not included for further testing. As a result, rs17704680 showed a G×multiE interaction (*p* = 3.01 × 10^–3^), that satisfied the threshold of significance (*p* = 1.67 × 10^–2^ = 0.05/3), whereas rs345749 showed no significant interaction (*p* = 1.45 × 10^–1^) (Supplementary Table S15). We also summarized the results of BF and G×singleE interaction effects of the 2 SNPs in HEXA (Supplementary Table S16). Among 4 factors, the best BF factor for rs17704680 was attributed to PWI (BF = 2.04) which differed from the result using the UKB samples with the BF of BMI showing the highest BF value. This may be related to differences in factors such as the ethnicity and environmental factors between the first G×multiE interaction analysis and replication test.

## Discussion

In this study, we performed two types of genome-wide G×E interaction studies of late-onset asthma in European individuals. The environmental factors used in this study included alcohol intake frequency, BMI, insomnia, MET score, neuroticism score, time spent watching TV, and TDI. For G×singleE interactions, we did not detect any genome-wide significant SNPs (*p* < 5.00 × 10^–8^/7 = 7.14 × 10^–9^). However, in the G×multiE interaction study, we found three independent genome-wide significant SNPs: rs117996675, rs345749, and rs17704680. We further identified the most relevant environmental factor per individual variant via BF values.

A recent study estimated the significance of G×E interactions using the polygenic risk score for obesity-related traits from the UKB and found that the G×E effect of GWAS SNPs significantly contributed to the phenotype variance of BMI by 1.9% in addition to the 15% of polygenic risk score effect (Sulc et al., 2020). Another report proposed a Bayesian whole-genome regression model for joint modeling of the main genetic effects and G×E interactions in large-scale datasets, known as Linear Environment Mixed Model Analysis (Kerin and Marchini, 2020). This analysis estimates a linear combination of environmental variables, known as an environment score, which interacts with genetic variants and can be used to estimate phenotypic variance attributable to G×E interaction effects. Using the UKB data, they estimated that for traits such as BMI, systolic blood pressure, and diastolic blood pressure, 9.3, 3.9, and 1.6%, respectively, of phenotypic variance is explained by G×E interactions and 27.4, 25.1, and 25.4% is explained by the main genetic effects (Kerin and Marchini, 2020). Despite some differences in the proportions of phenotypic variance, the reports mentioned above consistently suggested that the G×E interaction effects are proportionately smaller than the main genetic effects but were significant. Notably, Kerin and Marchini. (2020) suggested that low-frequency variants explain most of this phenotypic variance explained by G×E interactions (Kerin and Marchini, 2020). For asthma or related traits, such as hay fever and eczema, the phenotypic variance explained by G×E interaction effects is unclear.

The development of childhood-onset (or early-onset) asthma is often attributable to perinatal factors, atopy, viral respiratory-tract infections, and the microbiome (Ortqvist et al., 2009; Tedner et al., 2012; Arking et al., 2014; Lynch et al., 2017), whereas adult-onset (or late-onset) asthma is more strongly associated with obesity, smoking, and other environmental and occupational exposures (Amelink et al., 2013; Ilmarinen et al., 2015). Despite the lower genetic heritability of adult-onset asthma (*h* ^2^_g_ = 10.6%) than of childhood-onset asthma (*h* ^2^_g_ = 25.6%) (Ferreira et al., 2019), the high prevalence of adult-onset asthma suggests that environmental factors make a greater contribution to the development of asthma in adults than in childhood.

Functional annotations for the three novel loci suggest that variations in these SNPs are involved in immunological pathways. The SNP (rs117996675) near *PC* contains eQTLs such as *RIN1* (whole blood), *BRMS1* (stomach), and *ACTN3* (skeletal muscle and thyroid), which are all involved in immune system regulation. RIN1 (Ras and Rab interactor 1) is an RAS effector that binds to ABL1 and BCR-ABL1, promoting the cytoskeletal remodeling properties of ABL proteins and serves in part to regulate epithelial cell functions, including adhesion and migration (Hu et al., 2005). Interestingly, the expression of ABL was upregulated in the airway tissues of a mouse model of asthma and was associated with airway hyper-responsiveness in patients with severe asthma (Hu et al., 2005; Cleary et al., 2013). Our findings and previous studies indicate that allelic variations in rs117996675 affect airway remodeling by ABL through different expression of *RIN1* in late-onset asthma. BRMS1 (breast cancer metastasis suppressor 1) is associated with nuclear factor kappa light chain enhancer of activated B cells (NF-κB) that is highly related to inflammation and allergic diseases and promotes immunity by controlling the expression of genes involved in inflammation (Liu et al., 2006; Baker et al., 2011). The results from patients with severe and moderate asthma suggest that NF-κB protein is increased in several cell types of patients with asthma compared to in healthy subjects (Gagliardo et al., 2003; Edwards et al., 2009). This information supports that the regulation of *RIN1* and *BRMS1* is involved in the disease progression of asthma via allelic variations in rs117996675.

Meanwhile, the *ACTN3* locus (index SNP: rs540874, *r* ^2^ = 0.03 for rs117996675) was reported as a significantly shared locus (*P*_meta_ = 4.09 × 10^–9^) by meta-analysis for cross-trait associations between non-atopic asthma and BMI, indicating that our SNP (rs117996675) interacts with BMI to affect asthma, likely through *ACTN3* (Zhu et al., 2020). Although no information was available in the eQTL resource, *PC* (pyruvate carboxylase), containing rs117996675 in its intron, has been established as an important regulator of hepatic gluconeogenesis and tricarboxylic acid cycle function (Kumashiro et al., 2013; Cappel et al., 2019). Studies of animal models with loss-of-function for *PC* demonstrated that the loss of hepatic PC prevented insulin resistance and glucose intolerance, as well as reduced plasma lipid concentrations during feeding of a high-fat diet, suggesting an important contribution of hepatic PC to hyperglycemia during obesity (Kumashiro et al., 2013; Cappel et al., 2019). This information for the locus (rs117996675 in the 11q13.2) supports that BMI is a relevant effector on asthma, as evidenced by the BF values.

For rs345749, we found its eQTL signal in the *FMN1* locus in the thyroid, pituitary, and esophageal mucosa. *FMN1* has been reported to play a role in forming the adherens junction and in the polymerization of linear actin cables (Kobielak et al., 2004). *FMN1* expression is often dysregulated in the immune system such as in CD8 T-cells from pediatric patients with influenza-like illness or monocytes from patients with tuberculosis-associated immune reconstitution inflammatory syndrome, indicating its role in the immunological pathway (Tran et al., 2014; Henrickson et al., 2018). The polymorphism of rs345749 may be associated with the immune system via regulation of *FMN1* expression. The findings from BF values of rs345749 suggest that insomnia and alcohol intake frequency are the most relevant environments to asthma. Insomnia (or sleep deprivation) and asthma are associated, as 58% of patients with asthma reported impaired sleep quality (Braido et al., 2021). A study of an allergic mouse model showed that airway inflammation is more severe in allergic mice with sleep deprivation than in mice with healthy sleep, and inflammation in allergic mice with sleep deprivation is marked by an influx of neutrophils (mainly) and eosinophils and secretion of IL-6, TNF-α, and IL-17 compared to the eosinophilic inflammation and IL-4 production observed in allergic mice with healthy sleep (Nunes et al., 2018). These reports support insomnia as a relevant environmental interactor in asthma, although it remains unknown how our SNP, rs345749, participates in this interaction.

The rs17704680 in the 16p12.1 has a weak pQTL signal with TSLP (Sun et al., 2018). TSLP is a cytokine primarily expressed by the airway epithelium and released in response to environmental insults, inducing a range of downstream inflammatory processes (Varricchi et al., 2018; Gauvreau et al., 2020). The expression of TSLP is increased in the airways of patients with asthma compared to in healthy individuals, and is correlated with disease severity and lung function; polymorphisms in *TSLP* are associated with asthma (Gauvreau et al., 2020). This data indicates that regulation of *TSLP* via rs17704680 is involved in the development or/and process of asthma.

In the interaction study, we found that for 2 SNPs (rs117996675 and rs17704680), BMI was the most relevant environment. Previous association studies of obesity with asthma suggested that obesity-associated asthma consists of two forms of severe asthma, one form (late-onset) with a non-type 2 phenotype and the other form (early-onset) with a variation of type 2 asthma, complicated by the development of obesity (Dixon et al., 2010; Umetsu, 2017). These reports suggest that different types of asthma develop depending on the obesity status, supporting BMI as a possible interactor in asthma.

The strengths of the present interaction study are as follows: a moderately large-sized sample, the robust power of StructLMM, simultaneous incorporation of multiple factors, and prioritization of relevant factors. Genome-wide interaction studies require a large sample size to avoid false-positive findings and to detect small effect sizes (Turner, 2017). For asthma or asthma-related traits, many studies were underpowered in the sample size. Our current interaction study includes the largest number of cases (*N* = 14,089) of late-onset asthma and controls (*N* = 139,706) evaluated to date (Turner, 2017; Morales and Duffy, 2019). Despite this sample size, G×singleE interaction analyses did not produce a genome-wide significant signal, indicating that the power threshold for detecting interactions was not reached. However, StructLMM yielded three genome-wide significant loci for asthma that interact with up to seven environmental factors, indicating a superior detection power of this model compared with conventional genome-wide interaction analysis via PLINK. Moore et al. (2019) proposed this structured linear mixed model StructLMM to identify loci that interact with one or more environments and demonstrated successful application of StructLMM to BMI in the UKB (252,188 individuals), where this model validated previously known G×E interaction signals and moreover produced genome-wide significance (*p* < 5.00 × 10^–8^) for detecting novel interaction loci (Moore et al., 2019). Polimanti et al. (2021) reported a strong association of lifetime polysubstance dependence with lifetime suicidality and using StructLMM, identified multivariate G×E interaction loci (*LCAT*, *p* = 1.82 × 10^–7^; *TSNAXIP1*, *p* = 2.13 × 10^–7^; *CENPT, p* = 2.32 × 10^–7^; *PARD6A*, *p* = 5.57 × 10^–7^) of suicidality that interact with 4 substance dependences such as opioid, cocaine, nicotine, and polysubstance dependences (15,557 American participants in the Yale-Penn cohort) (Polimanti et al., 2021). We previously applied this StructLMM to BMI in 8,155 Korean samples from the Korea Association Resource to evaluate interactions with seven factors including alcohol consumption, education, income, total calorie intake, protein intake, carbohydrate intake, and smoking status (Jung et al., 2021). This previous study using StructLMM identified genome-wide significant interaction loci (rs2391331 in the *EFNB2*; *p* = 5.03 × 10^–10^) with BMI, and BF analyses showed that six environmental factors, except for carbohydrate intake, contributed to the interaction with this SNP on BMI (Jung et al., 2021).

An advantage of this StructLMM is that it incorporates multiple environments as additive, random effect components into an interaction test. Therefore, StructLMM accounts for simultaneous interactions between various environments and a trait. In this study, we employed seven environmental factors previously shown to be associated with asthma and were associated with asthma in the UKB samples. By estimating the BF values, we prioritized environmental factors for individual variants. For rs117996675, BMI was the most relevant environment as evidenced by BF values of StructLMM and by *p*-values of the G×singleE interaction. These results were partially supported by functional and regional annotations such as its associations with *ACTN3* by eQTL and its location in the *PC* locus.

This study also had some limitations. First, the study was based on a cross-sectional dataset, presenting the possibility of reverse causality. To limit this design bias, we focused on late-onset asthma (onset age of ≥16 years; mean onset age of 39.8 years) rather than on early-onset or total asthma. The individuals included in this study were limited to 40–69 years old. Moreover, the association directions between asthma and six factors (BMI, insomnia, MET score, neuroticism score, time spent watching TV, and TDI) correspond to the findings of previous studies (Hedlund et al., 2006; Eijkemans et al., 2012; Meltzer et al., 2014; Vancampfort et al., 2017; Kavanagh et al., 2018; Najjab et al., 2020), suggesting reasonably acceptable associations. Several studies have used disease outcomes from a cross-sectional dataset for interaction analysis and derived important results (Eze et al., 2016; Jia et al., 2021). Next, some environmental data in the present study are from self-reported questionnaires (e.g., alcohol intake frequency, insomnia, MET score, neuroticism score, time spent watching TV, and TDI) that are prone to responder bias (Rask-Andersen et al., 2017). Thus, the presence of an interviewer is recommended to reduce the likelihood of responder bias when obtaining self-reported questionnaire data.

Next, the StructLMM is based on linear mixed models (LMM) which are not designed to analyze binary traits and can have inflated type I error rates, especially in the presence of unbalanced case-control ratios. A score test called the generalized linear mixed model (GLMM) has been developed to fit logistic regression of binary traits.(Chen et al., 2016; Zhou et al., 2018). Unfortunately, we could not adapt the StructLMM methods into such GLMM-based methods and are not aware of such reports yet. However, we found that there is an acceptable common practice when using LMM-based methods for binary traits. To avoid the type I error inflation, a recommended practice is to remove rare variants (for example, MAF <0.01) and phenotypes with highly unbalanced case-control ratios (for example, <1:100).(Chen et al., 2016; Loh et al., 2018; Zhou et al., 2018; Jiang et al., 2019; Jiang et al., 2021). For our study, the case-control ratio for asthma was 1:10 that is considered a balanced binary trait and we tested common variants with MAF >0.01 in white British individuals of the UKB. Therefore, we suggest that StructLMM is still applicable for binary traits such as asthma with some limitations.

Finally, we attempted two sets of replications using Europeans that were unused in the discovery set and East Asian samples. Interestingly, rs17704680 showed a nominally significant G×multiE interaction in both replication samples. Moreover, in the replication set of European samples, the best BF factor for rs17704680 was attributed to BMI, consistently supporting the discovery result of European samples. However, because of the limited availability of the factors surveyed in East Asian samples, we used only 4 environmental factors and replaced 2 of these factors with similar factors (neuroticism score by PWI; TDI by total household income). Thus, the results obtained for East Asian samples may not strongly support these interactions. Therefore, our findings imply that the replication for G×multiE interactions may require the homogeneity between the replication sample and the discovery sample regarding environmental factors and allele frequencies. Hence, further validation is warranted using another asthma cohort.

In conclusion, we identified three novel, genome-wide interaction signals in late-onset asthma that interact with up to seven environmental factors. These three loci are associated with immune system regulation; additionally, rs117996675 may primarily interact with BMI to affect late-onset asthma. Although further confirmation is needed, our findings improve the understanding of the role of the immune system and genetic interactions with environmental exposures in late-onset asthma and may be applied in precision treatment of asthma.

## Data Availability

Publicly available datasets were analyzed in this study. The names of the repository/repositories and accession number(s) can be found in the article/Supplementary Material.

## Abbreviations

G×singleE, gene-by-single environment; G×multiE, gene-by-multi-environment; StructLMM, structured linear mixed model; BMI, body mass index; MET, metabolic equivalent of task; TDI, Townsend deprivation index; UKB, United Kingdom Biobank; PC, principal component; COPD, chronic obstructive pulmonary disease; FEV1, forced expiratory volume in 1-s; BF, Bayes factor; HEXA, Health Examinees; KoGES, Korean Genome and Epidemiology Study; PWI, psychological well-being index; CADD, Combined Annotation-Dependent Depletion; TSLP, thymic stromal lymphopoietin; NF-κB, nuclear factor kappa light chain enhancer of activated B; PC, pyruvate carboxylase.
