# Supplementary material for: Genome-Wide Interaction Study of Late-Onset Asthma With Seven Environmental Factors Using a Structured Linear Mixed Model in Europeans
Source: Front Genet. 2022 Mar 30;13:765502. doi: 10.3389/fgene.2022.765502 (PMC9005993; doi:10.3389/fgene.2022.765502)
Supplement: Supplementary file 1 [file Presentation1.PPTX]

## Slide 1
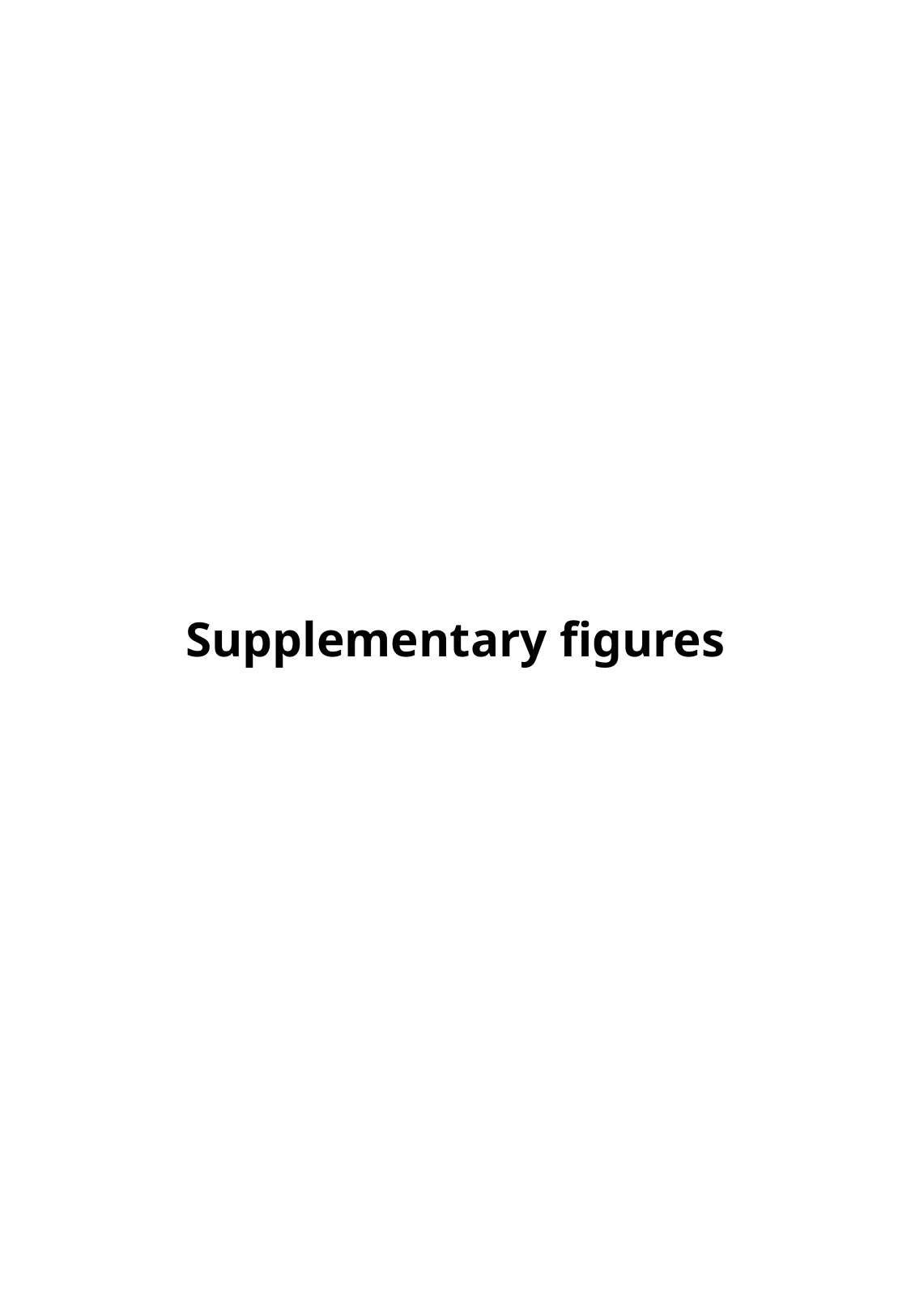

# Supplementary figures

## Slide 2
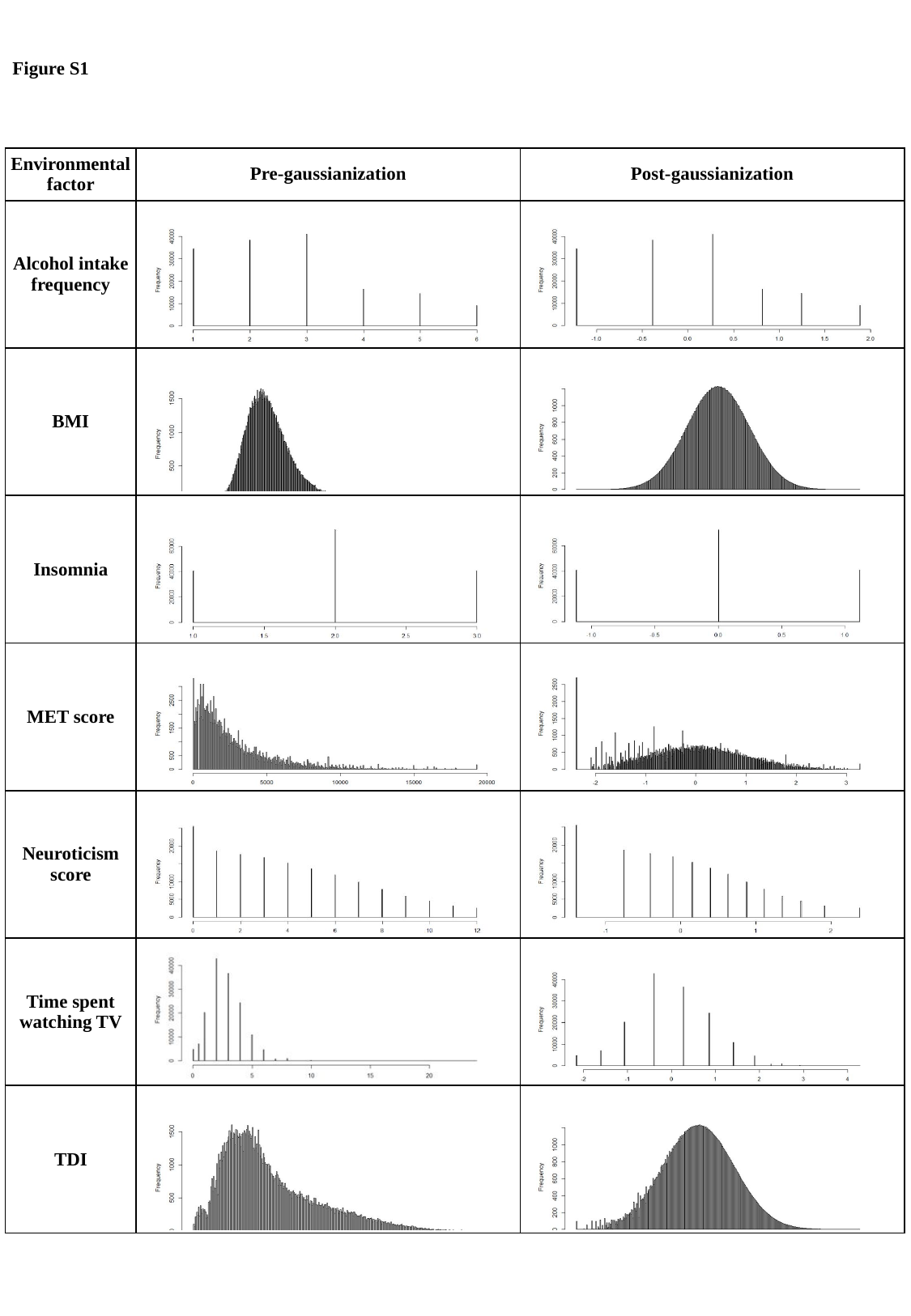

Figure S1
| Environmental factor | Pre-gaussianization | Post-gaussianization |
| --- | --- | --- |
| Alcohol intake frequency | | |
| BMI | | |
| Insomnia | | |
| MET score | | |
| Neuroticism score | | |
| Time spent watching TV | | |
| TDI | | |

## Slide 3
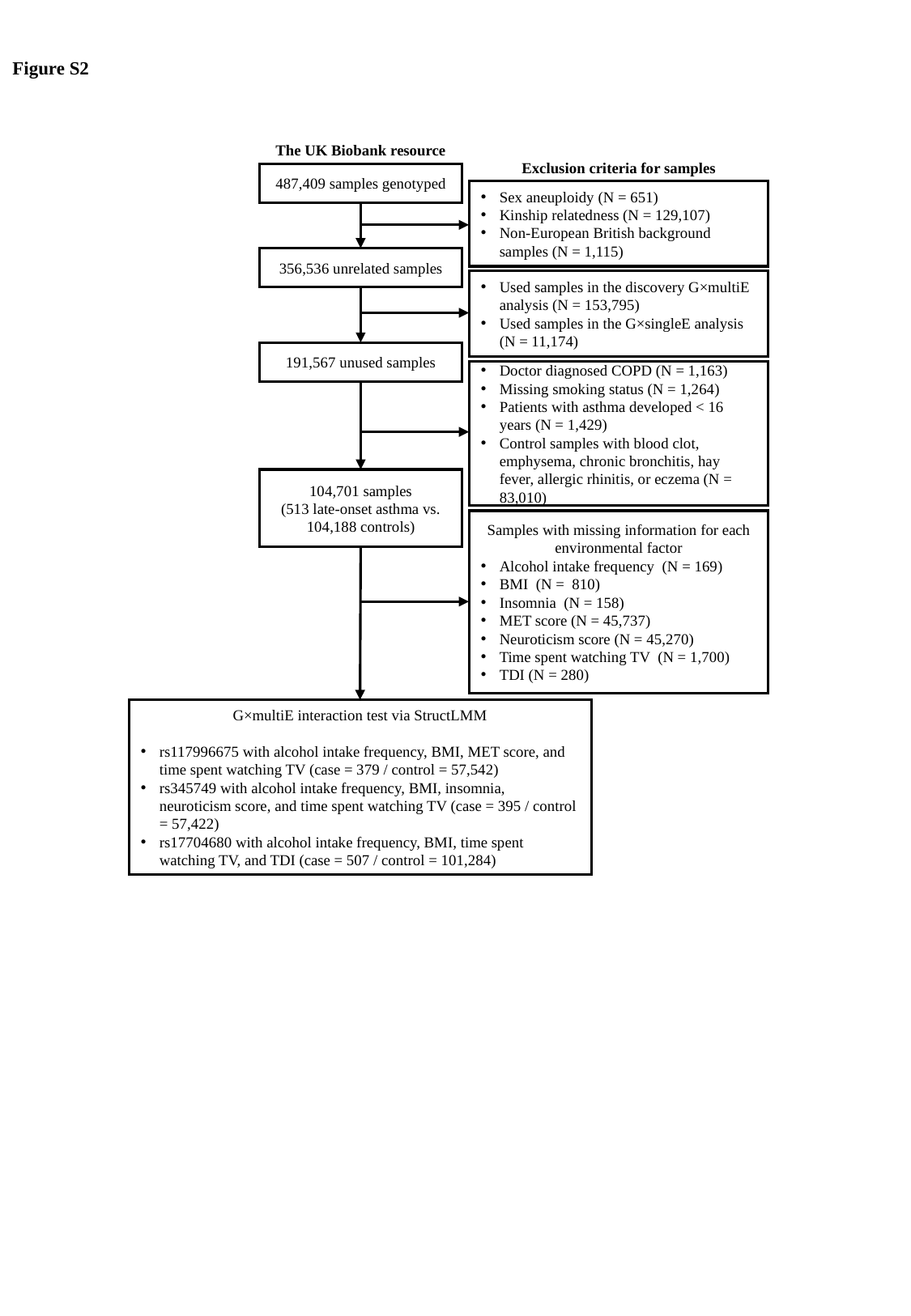

Figure S2
The UK Biobank resource
Exclusion criteria for samples
487,409 samples genotyped
Sex aneuploidy (N = 651)
Kinship relatedness (N = 129,107)
Non-European British background samples (N = 1,115)
356,536 unrelated samples
Used samples in the discovery G×multiE analysis (N = 153,795)
Used samples in the G×singleE analysis (N = 11,174)
191,567 unused samples
Doctor diagnosed COPD (N = 1,163)
Missing smoking status (N = 1,264)
Patients with asthma developed < 16 years (N = 1,429)
Control samples with blood clot, emphysema, chronic bronchitis, hay fever, allergic rhinitis, or eczema (N = 83,010)
104,701 samples
(513 late-onset asthma vs.
104,188 controls)
Samples with missing information for each environmental factor
Alcohol intake frequency (N = 169)
BMI (N = 810)
Insomnia (N = 158)
MET score (N = 45,737)
Neuroticism score (N = 45,270)
Time spent watching TV (N = 1,700)
TDI (N = 280)
G×multiE interaction test via StructLMM
rs117996675 with alcohol intake frequency, BMI, MET score, and time spent watching TV (case = 379 / control = 57,542)
rs345749 with alcohol intake frequency, BMI, insomnia, neuroticism score, and time spent watching TV (case = 395 / control = 57,422)
rs17704680 with alcohol intake frequency, BMI, time spent watching TV, and TDI (case = 507 / control = 101,284)

## Slide 4
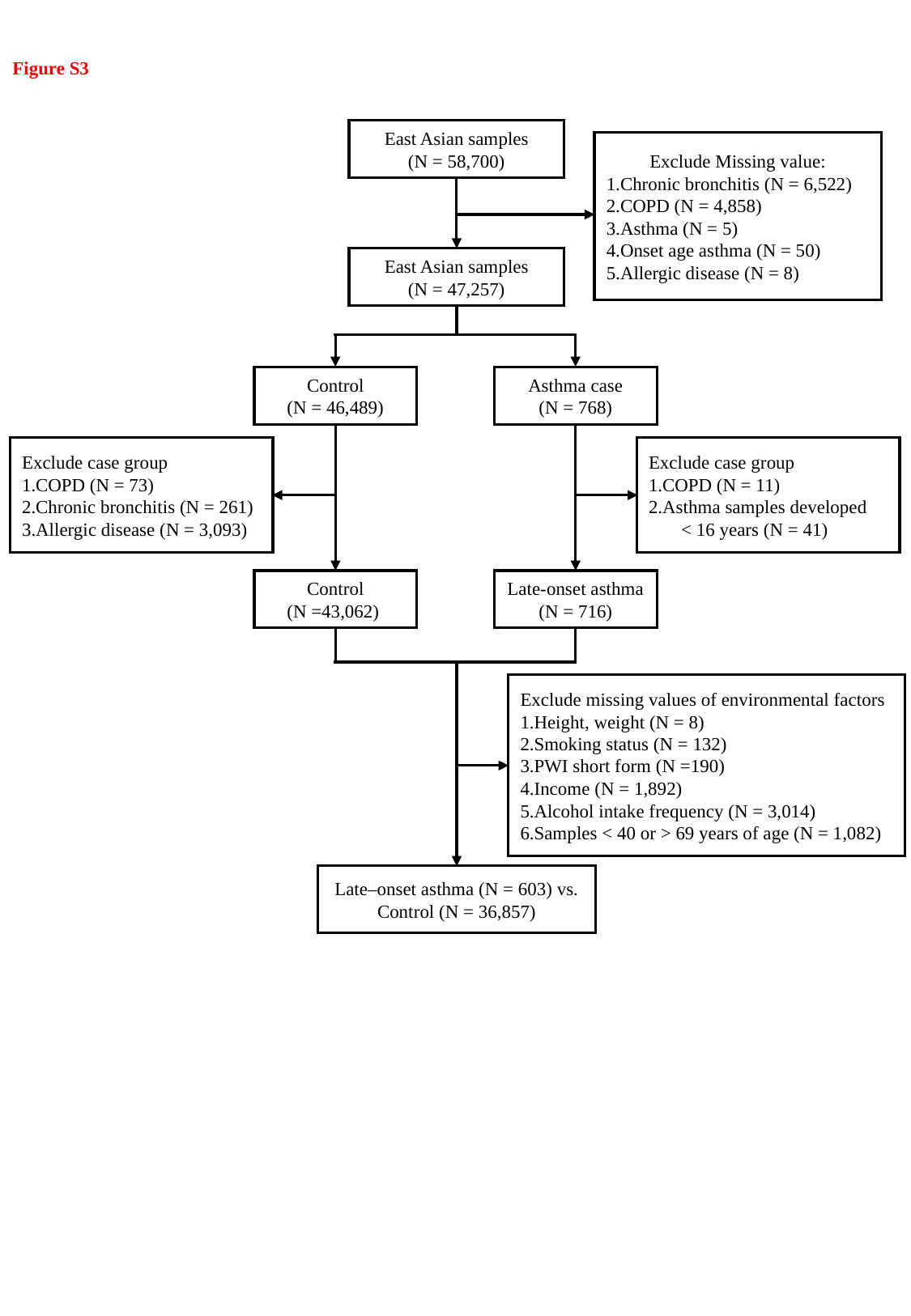

Figure S3
East Asian samples
(N = 58,700)
Exclude Missing value:
Chronic bronchitis (N = 6,522)
COPD (N = 4,858)
Asthma (N = 5)
Onset age asthma (N = 50)
Allergic disease (N = 8)
East Asian samples
(N = 47,257)
Control
(N = 46,489)
Asthma case
(N = 768)
Exclude case group
COPD (N = 73)
Chronic bronchitis (N = 261)
Allergic disease (N = 3,093)
Exclude case group
COPD (N = 11)
Asthma samples developed
 < 16 years (N = 41)
Control
(N =43,062)
Late-onset asthma
(N = 716)
Exclude missing values of environmental factors
Height, weight (N = 8)
Smoking status (N = 132)
PWI short form (N =190)
Income (N = 1,892)
Alcohol intake frequency (N = 3,014)
Samples < 40 or > 69 years of age (N = 1,082)
Late–onset asthma (N = 603) vs. Control (N = 36,857)

## Slide 5
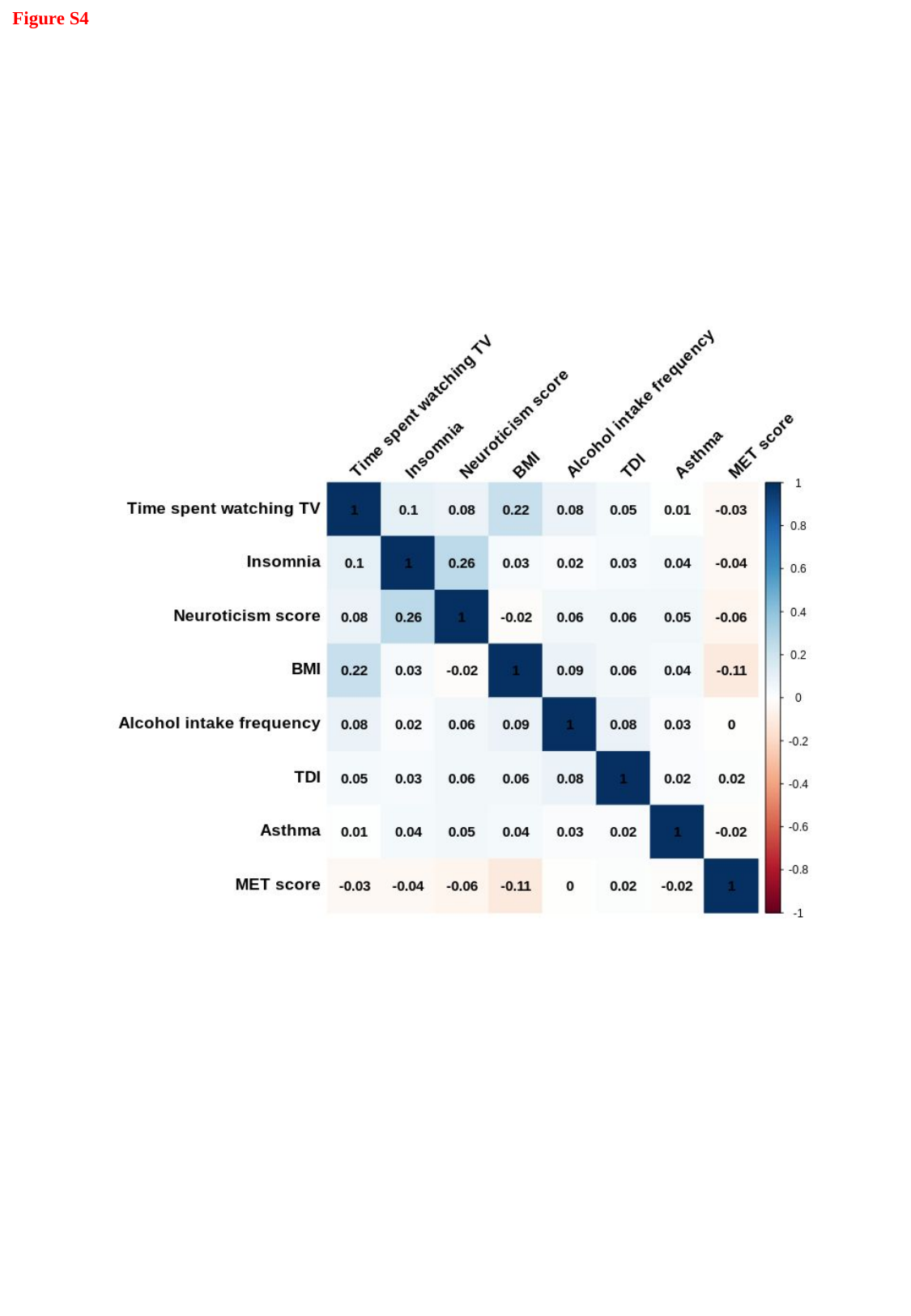

Figure S4

## Slide 6
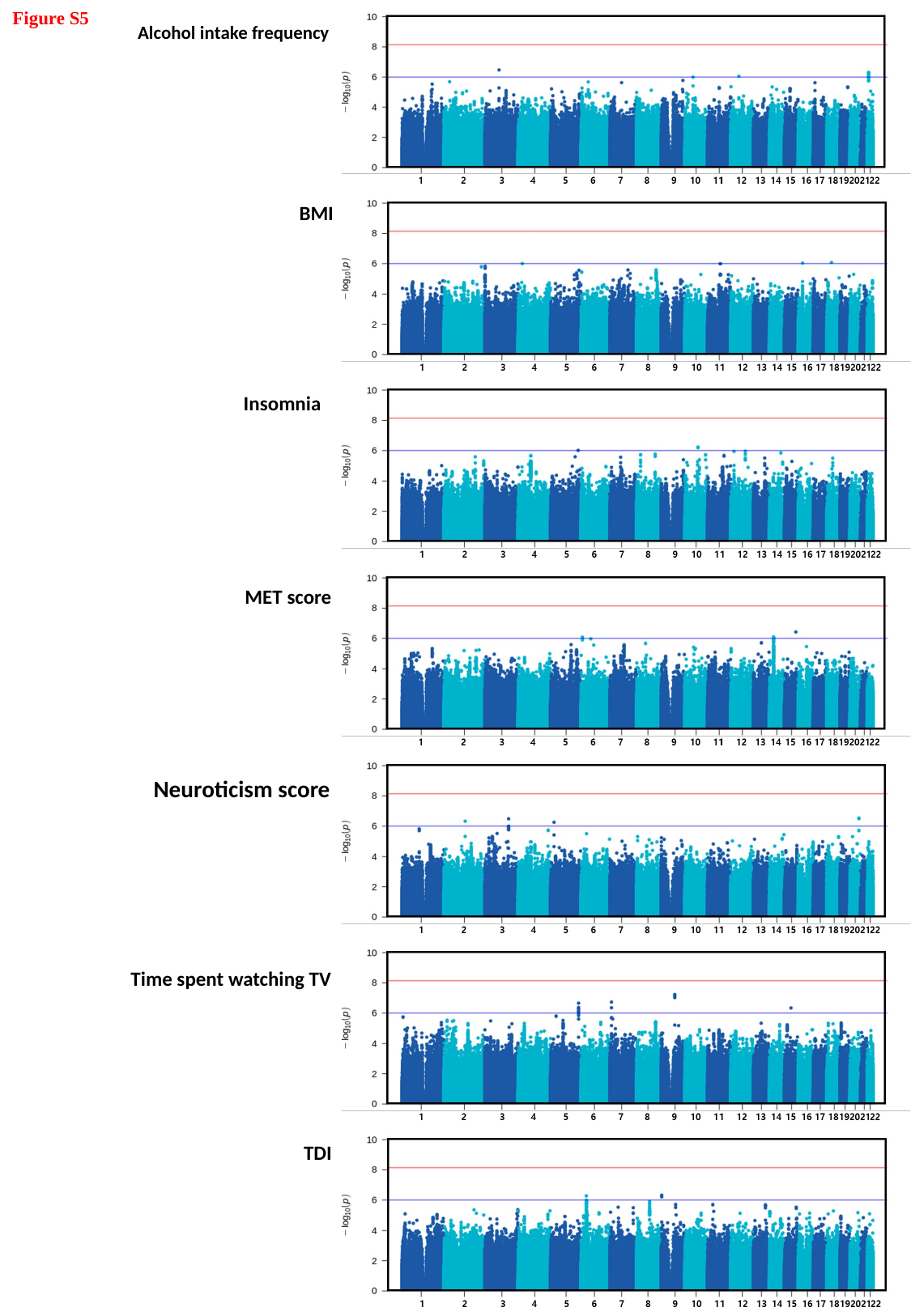

Figure S5
Alcohol intake frequency
BMI
Insomnia
MET score
Neuroticism score
Time spent watching TV
TDI
